# Association between the systemic immune-inflammatory index and the immune response after hepatitis B vaccination: a cross-sectional analysis of NHANES data

**DOI:** 10.3389/fpubh.2025.1480766

**Published:** 2025-05-14

**Authors:** Jiayan Hu, Kaiyue Huang, Hede Zou, Junxiang Li

**Affiliations:** ^1^Dongfang Hospital, Beijing University of Chinese Medicine, Beijing, China; ^2^Beijing University of Chinese Medicine, Beijing, China; ^3^Xiyuan Hospital of China Academy of Chinese Medical Sciences, Beijing, China

**Keywords:** National Health and Nutrition Examination Survey, systemic immune-inflammatory index, hepatitis B, vaccine, antibody

## Abstract

**Aim:**

Our research aimed to investigate the relationship between the systemic immune-inflammatory index (SII) and the immunological response to hepatitis B vaccination.

**Methods:**

We collected data from the National Health and Nutrition Examination Survey database from 2007 to 2018. To examine the association between the SII and immunological response, we conducted weighted multiple regression analysis and subgroup analysis. Furthermore, we utilized restricted cubic splines (RCSs) to analyze the linear relationship between the two variables.

**Results:**

In our study, we included a total of 6,123 patients, of whom 2,770 tested positive for hepatitis B antibodies. Multivariate logistic regression analysis indicated that, after controlling for all measured factors, a high level of the SII was inversely associated with the presence of antibodies following three doses of the hepatitis B vaccine (OR = 0.8661, 95% CI = 0.7577–0.9899, *p* = 0.035). Subgroup analysis and interaction testing revealed that sex, age, body mass index, diabetes, and other factors did not significantly influence this negative association (P for interaction >0.05). Additionally, the RCS model revealed no non-linear relationship between the SII and the immune response to the hepatitis B vaccine (*p* > 0.05). Notably, antibody expression significantly decreased as the SII increased beyond the threshold of 448.3.

**Conclusion:**

This cross-sectional study revealed a strong association between low antibody production following hepatitis B vaccination and the SII. However, this cross-sectional study could not establish a causal relationship between the two variables. Therefore, further experimental verification is necessary to confirm the correlation observed in our study.

## 1 Introduction

Hepatitis B is a liver infection caused by the hepatitis B virus (HBV). The global prevalence of hepatitis B was 3.2% in 2022, corresponding to 257.5 million (216.6–316.4) individuals positive for HBsAg (1). Chronic hepatitis B (CHB) is a significant public health concern because it can lead to liver cirrhosis and hepatocellular carcinoma (HCC), potentially resulting in death. A survey of the global burden of hepatitis B indicated that the number of deaths attributed to the disease worldwide reached 523,003 in 2019, with projections suggesting that this figure could rise to 628,824 by 2030 (2). China accounts for a large part of the global burden of HBV infection, playing a pivotal role in achieving the WHO 2030 global hepatitis elimination target. A meta-analysis that included 3,740 studies and 231 million subjects revealed that the HBsAg seroprevalence in the general population of China decreased from 9.6% in 1973–1984 to 3.0% in 2021 (3). A significant development in the fight against hepatitis B viral infection is the development of hepatitis B vaccination. The physiological immune response to vaccination involves a complex interplay of various immune cells, including B cells, T cells, and antigen-presenting cells. Upon vaccination, the body recognizes the introduced antigens and activates these immune cells, leading to the production of antibodies and the establishment of immunological memory. This response is influenced by several factors, including the individual’s age, health status, and underlying inflammatory conditions (4). Reports claim that if two or three doses are given, the protection rate can exceed 90% (5). However, 5% of individuals with normal immune function do not respond to the vaccine (6). Uncertain genetics, chronic illnesses, and the use of immune modulators may all play roles in this lack of response.

Numerous different biomarkers, including immunoglobulins, complement, and C-reactive protein, have been employed to assess the immunological and inflammatory status of various diseases. At present, hematological parameters, including the neutrophil-to-lymphocyte ratio (NLR), platelet-to-lymphocyte ratio (PLR), and systemic immune-inflammatory index (SII), have become new biomarkers for the diagnosis, prognosis, risk stratification and prediction of survival and mortality in cardiovascular diseases (7), autoimmune diseases (8), and parasitic diseases (9). The SII levels are determined by the following equation: platelet count × neutrophil count/lymphocyte count (10, 11). The important role of lymphocytes in immune and inflammatory responses is widely recognized (12). Neutrophils are the core effector cells of innate immunity, directly eliminating pathogens through phagocytosis, releasing antimicrobial proteins, and forming neutrophil extracellular traps (NETs) while activating adaptive immune signals (13). Platelets not only participate in hemostasis but also recruit and activate neutrophils and lymphocytes by adhering to pathogens and releasing immune-modulatory molecules while promoting vascular permeability to promote immune cell infiltration. Together, these three components form a dynamic network: neutrophils respond rapidly to infections, platelets bridge innate and adaptive immunity, and lymphocytes achieve precise targeting, collectively maintaining immune defence and inflammatory balance (14). It is hypothesized that the SII represents the equilibrium between immune responses that fight tumors, such as T-cytotoxic cells, and immunological responses that promote tumor growth (15). At present, the interaction between systemic inflammation and the local immune response has been identified as the seventh cancer hallmark and has been shown to be related to the occurrence, development, and progression of various types of cancer (16–18). In addition, an elevated SII was found to be an important risk factor for non-neoplastic diseases. As Song et al. reported, the SII is positively correlated with increased hepatic steatosis (19). Overall, a high serum SII is associated with poor prognosis, a shorter recurrence time, and reduced overall survival.

Immune dysfunction is reflected by signs of systemic inflammation. However, the relationship between the SII and antibody expression following hepatitis B vaccination has not been studied. Given the high incidence of HBV infection and the current situation in which some individuals have no effective response to the hepatitis B vaccine, we conducted a population-based cross-sectional study to investigate the relationship between the SII of adult participants in the National Health and Nutrition Examination Survey (NHANES) and the immune response after hepatitis B vaccination.

## 2 Materials and methods

### 2.1 Study population

The data in this article are from the NHANES, a nationally representative cross-sectional survey designed and implemented by the National Center for Health Statistics (NCHS). The survey uses a stratified, multistage probability method to sample the U.S. population and provides health and nutrition statistics of the U.S. non-institutional civilian population. The NCHS Research Ethics Review Board authorized the investigation and verified that all participants provided informed consent. The detailed statistics are available at https://www.cdc.gov/nchs/nhanes/.

**Figure 1:**
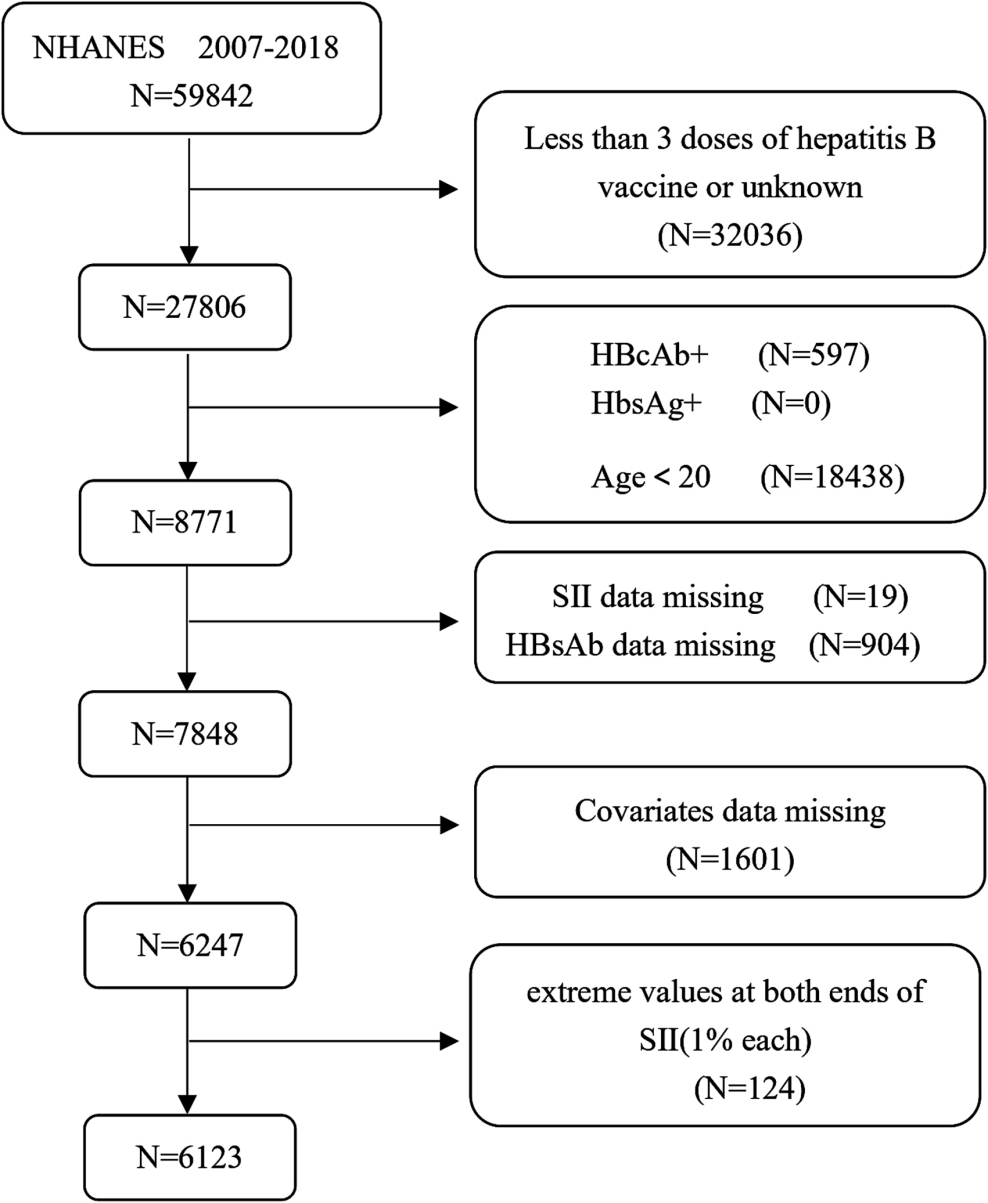
A flowchart of participant selection.

A total of 59,842 participants’ data from the NHANES from 2007 to 2018 were included. Only participants who had received at least three doses of the hepatitis B vaccine were included because the seroprevalence of anti-HBs was lower in those who had received fewer than three doses. We included adults (≥20 years old) who had received three doses of the hepatitis B vaccine, whose hepatitis B serum status was recorded following vaccination, and who were free of both current (*n* = 0; hepatitis B surface antigen positivity) and past (*n* = 597; hepatitis B core antibody positivity) HBV infection. We excluded 19 participants with missing SII data, 904 with missing HBsAb data and 1,601 with missing variables (to be discussed later). At the same time, we deleted 1% of the extreme values from both ends of the SII to prevent the impact of extreme values on the results. This method has been used multiple times in previous studies (20). Finally, this study included 6,123 participants. The flowchart is shown in Figure 1.

### 2.2 Assessment of hepatitis B serostatus

This study used VITROS anti-HBs, anti-HBc, and hepatitis B surface antigen detection (Ortho Clinical Diagnosis Company, Lalitan, New Jersey, USA), and it adopted the standardized calibration and quality control scheme described on the NHANES website.^1^ The detection range of anti-HBs is 4.23–1,000 mIU/mL. A value of <5.00 mIU/mL is negative, and a value of ≥12.0 mIU/mL is positive (21). Samples with initial results >5.00 mIU/mL and <12.0 mIU/mL were repeated two times. If the two repeats were <5.00 mIU/mL, the sample was reported as negative. If ≥12.0 mIU/ml was detected in two repeated tests, the sample was reported as positive. The outcome was ambiguous if one or more duplicate results fell between 5.00 mIU/mL and 12.0 mIU/mL. According to the NHANES database, only anti-HB levels of 12.0 mIU/mL or higher were considered positive. Thus, if any repeat results were between 5.00 mIU/mL and 12.0 mIU/mL, those subjects would be classified as negative and excluded from this study.

### 2.3 SII and covariates

SII levels were determined by the following equation: platelet count × neutrophil count/lymphocyte count (10, 11). Lymphocyte, neutrophil, and platelet counts were measured using an automated hematological analysis device (UniCel DxH800 analyzer, Beckman Coulter, Inc.) and reported as 10^3^ cells/ml. According to previous studies, possible confounding factors related to the SII and hepatitis B vaccine response were included in the final analysis (22). The covariates included in our study that may have affected the results included age, sex, and race (Mexican American, White Non-Hispanic, Black Non-Hispanic, other Hispanic, other races), education (under high school, high school or equivalent, college graduate or above), poverty-to-income ratio (PIR < 1.5, 1.5–3.5, >3.5), body mass index (≤18.5 kg/m^2^, 18.5–25, 25–30, >30), marital status (non-single, single), smoking (never, former, current), alcohol use (never, former, mild, moderate, heavy), diabetes (yes, no), and creatinine level.

### 2.4 Statistical analysis

The SII was divided into tertiles from the lowest (Q1) to the highest (Q3). Specifically, after excluding extreme outliers (trimming the top/bottom 1% of the SII values), we categorized participants into tertiles (Q1–Q3) using cohort-specific 33.3rd/66.6th percentiles of the trimmed SII distribution. Tertiles were analyzed as ordinal variables in regression models to evaluate dose–response effects on seropositivity. We used the survey-weighted mean (95% CI) (continuous variable) and survey-weighted percentage (95% CI) (categorical variable) to assess the difference in whether subjects grouped by the SII tertile were positive for anti-HBS. Multivariate logistic regression analysis was used to test the correlation between the SII and positive HBsAb expression, and Model 1 was adjusted for age, sex, and race. Model 2 was adjusted for age, sex, race, income-to-poverty ratio, education level, drinking status, smoking status, BMI, and diabetes status. The results are expressed as odds ratios (ORs) and 95% confidence intervals (CIs). Restricted cubic splines (RCSs) were used to explore non-linear relationships. Finally, we performed subgroup analysis according to sex, age, race, and other patient differences. All significance tests were two-sided, and *p* < 0.05 was used as the significance level.

## 3 Results

### 3.1 Baseline characteristics of participants

This study included 6,123 individuals with an average age of 39.85 years, including 2,671 men (43.6%). We classified the participants into two groups on the basis of whether they were positive for anti-HBS, and Table 1 displays the clinical characteristics of each group. After three doses of the hepatitis B vaccine, we discovered that women, young persons, those with high incomes, low BMIs, high levels of education, those who do not smoke, those who do not have diabetes, and those with low SII values were more likely to generate protective antibodies (12.0 mlU/mL). However, there was no difference in the creatinine level or marital status between the two groups. It is difficult to achieve sufficient anti-HBS titres in patients with advanced kidney disease, especially in hemodialysis patients (23, 24). In patients with renal failure, an increase in the serum creatinine level decreases the immunological response to the hepatitis B vaccine (24, 25). Therefore, we evaluated the correlation between the serum creatinine level and anti-HBS. Although there was no statistically significant difference between the two groups, our investigation indicated that the anti-HBS-negative group had a higher creatinine level. This disparity could be explained by a number of variables, including sample size and race.

**Table 1:** Weighted characteristics of the study population based on anti-HBS (*n* = 6,123).

	anti-HBS (−) *N* = 3,353	anti-HBS (+) *N* = 2,770	*p*-value
Age	41.679 (40.796–42.562)	36.888 (36.034–37.741)	<0.0001
PIR	2.954 (2.856–3.053)	3.242 (3.134–3.349)	<0.0001
BMI	30.161 (29.778–30.545)	27.950 (27.553–28.347)	<0.0001
Creatinine (Umol/L)	77.032 (75.738–78.326)	75.426 (74.343–76.510)	0.0647
SII	515.974 (504.371–527.576)	494.618 (481.923–507.314)	0.0093
Sex			<0.0001
Male	48.257 (45.869–50.653)	39.610 (37.368–41.897)	
Female	51.743 (49.347–54.131)	60.390 (58.103–62.632)	
Race			<0.0001
Mexican American	8.499 (6.773–10.615)	5.457 (4.438–6.693)	
Other Hispanic	11.515 (9.844–13.428)	9.656 (8.230–11.299)	
White Non-Hispanic	67.164 (63.466–70.660)	71.422 (68.214–74.428)	
Black Non-Hispanic	6.205 (4.981–7.706)	4.751 (3.702–6.079)	
Other Race	6.617 (5.713–7.653)	8.714 (7.295–10.378)	
Marital			0.1476
Non-single	62.021 (59.124–64.834)	59.372 (56.133–62.532)	
Single	37.979 (35.166–40.876)	40.628 (37.468–43.867)	
Education			<0.0001
<High school	2.687 (2.168–3.328)	0.955 (0.696–1.308)	
High school	30.638 (28.026–33.381)	18.338 (16.415–20.432)	
>High school	66.675 (63.768–69.459)	80.707 (78.557–82.689)	
Alcohol status			0.0028
Never	9.828 (8.223–11.706)	8.658 (7.118–10.494)	
Former	10.581 (9.248–12.081)	7.122 (5.869–8.618)	
Mild	35.486 (33.055–37.996)	37.124 (34.577–39.744)	
Moderate	19.287 (17.683–20.999)	21.823 (19.565–24.262)	
Heavy	24.817 (22.561–27.221)	25.273 (22.986–27.705)	
Smoke status			<0.0001
Never	57.401 (54.661–60.096)	65.498 (62.960–67.950)	
Former	22.016 (19.831–24.368)	17.936 (15.885–20.189)	
Now	20.584 (18.735–22.564)	16.566 (14.714–18.601)	
Diabetes			<0.0001
No	85.372 (83.565–87.011)	95.004 (93.981–95.862)	
Yes	14.628 (12.989–16.435)	4.996 (4.138–6.019)	
PIR categorical			<0.0001
<1	16.142 (14.402–18.047)	12.501 (10.869–14.338)	
≥1, <3	35.084 (32.829–37.408)	30.718 (28.045–33.527)	
≥3	48.774 (45.898–51.659)	56.781 (53.613–59.895)	
BMI categorical			<0.0001
≤18.5	1.211 (0.873–1.679)	2.004 (1.457–2.749)	
>18.5, ≤25	25.356 (23.088–27.765)	36.716 (33.847–39.683)	
>25, ≤30	30.823 (28.419–33.335)	30.211 (27.748–32.794)	
>30	42.610 (40.164–45.093)	31.069 (28.509–33.750)	
SII quartile			0.0091
Q1	31.565 (29.676–33.518)	32.886 (30.054–35.847)	
Q2	32.788 (30.653–34.997)	36.166 (33.689–38.718)	
Q3	35.647 (33.415–37.943)	30.948 (28.550–33.453)	

For continuous variables, the survey-weighted mean (95% CI) and the *p*-value were measured using the survey-weighted t-test.

For categorical variables, such as survey-weighted percentage (95% CI), the *p*-value was determined by the survey-weighted Chi-square test.

PIR, poverty-to-income ratio; BMI, body mass index; SII, systemic immune-inflammation index; Q, tertiles.

After the SII was measured, we further examined the participants’ clinical characteristics (Table 2). In terms of sex, the household income-to-poverty ratio, race, marital status, BMI, education level, alcohol intake, diabetes status, and surface antigen positivity, the SII tertiles revealed statistically significant differences (*p* < 0.05). The participants in the SII high-level group had a greater proportion of anti-HBS-negative individuals, and they were more likely to be female, have a low income, be unmarried, have a BMI of >30 kg/m^2^, be well educated, be heavily intoxicated, and be free of diabetes.

**Table 2:** Weighted characteristics of the study population based on SII trisection.

	Overall	Low	Middle	High	*p*-value
Age	39.339 (38.647–40.032)	38.900 (37.855–39.945)	39.622 (38.728–40.515)	39.472 (38.498–40.447)	0.4451
PIR	3.095 (3.005–3.184)	3.110 (2.981–3.240)	3.196 (3.079–3.312)	2.975 (2.837–3.114)	0.0234
BMI	29.082 (28.761–29.402)	27.657 (27.272–28.042)	29.036 (28.595–29.477)	30.504 (29.957–31.051)	<0.0001
Creatinine (Umol/L)	76.248 (75.401–77.094)	77.358 (76.350–78.365)	76.017 (74.525–77.509)	75.414 (73.857–76.972)	0.1142
Sex					<0.0001
Male	44.035 (42.340–45.744)	50.873 (47.946–53.794)	43.884 (40.970–46.840)	37.587 (34.859–40.397)	
Female	55.965 (54.256–57.660)	49.127 (46.206–52.054)	56.116 (53.160–59.030)	62.413 (59.603–65.141)	
Race					<0.0001
Mexican American	7.014 (5.744–8.538)	6.256 (4.994–7.810)	6.637 (5.321–8.250)	8.135 (6.313–10.423)	
Other Hispanic	10.608 (9.196–12.206)	15.179 (12.762–17.961)	8.681 (7.403–10.157)	8.181 (6.908–9.665)	
White Non-Hispanic	69.243 (66.130–72.190)	65.966 (61.477–70.186)	71.100 (67.888–74.113)	70.490 (66.791–73.938)	
Black Non-Hispanic	5.495 (4.475–6.731)	4.788 (3.645–6.267)	5.497 (4.308–6.990)	6.176 (4.803–7.908)	
Other Race	7.641 (6.697–8.706)	7.811 (6.522–9.329)	8.085 (6.815–9.566)	7.018 (5.866–8.377)	
Marital					0.0319
No	60.728 (58.271–63.131)	60.500 (56.751–64.128)	63.513 (60.182–66.719)	58.072 (54.812–61.264)	
Yes	39.272 (36.869–41.729)	39.500 (35.872–43.249)	36.487 (33.281–39.818)	41.928 (38.736–45.188)	
EDU					0.0269
<High school	1.842 (1.552–2.184)	1.577 (1.099–2.259)	2.033 (1.600–2.580)	1.900 (1.384–2.602)	
High school	24.632 (22.738–26.630)	25.242 (22.372–28.346)	22.003 (19.597–24.613)	26.758 (23.963–29.752)	
>High school	73.526 (71.419–75.531)	73.181 (69.927–76.203)	75.965 (73.239–78.494)	71.342 (68.320–74.184)	
Alcohol status					0.0049
Never	9.257 (7.854–10.881)	9.010 (7.260–11.132)	9.398 (7.571–11.611)	9.348 (7.615–11.428)	
Former	8.892 (7.897–9.999)	8.553 (7.031–10.368)	7.949 (6.819–9.247)	10.194 (8.486–12.199)	
Mild	36.286 (34.394–38.221)	39.586 (36.162–43.116)	37.043 (34.184–39.996)	32.317 (29.390–35.391)	
Moderate	20.525 (19.105–22.023)	20.839 (18.357–23.559)	20.793 (18.474–23.319)	19.946 (17.811–22.269)	
Heavy	25.040 (23.453–26.696)	22.012 (19.363–24.911)	24.818 (22.515–27.273)	28.194 (25.133–31.471)	
Smoke status					0.0595
Never	61.354 (59.335–63.335)	62.862 (59.906–65.726)	61.620 (58.666–64.491)	59.623 (56.419–62.747)	
Former	20.024 (18.300–21.866)	20.294 (17.756–23.092)	20.662 (18.060–23.530)	19.105 (16.649–21.827)	
Now	18.622 (17.176–20.160)	16.844 (15.037–18.819)	17.718 (15.592–20.065)	21.272 (18.792–23.983)	
Diabetes					<0.0001
No	90.075 (88.984–91.069)	93.309 (91.770–94.577)	90.664 (88.864–92.198)	86.345 (84.101–88.315)	
Yes	9.925 (8.931–11.016)	6.691 (5.423–8.230)	9.336 (7.802–11.136)	13.655 (11.685–15.899)	
PIR categorical					0.0189
<1	14.364 (12.951–15.903)	14.458 (12.411–16.778)	12.526 (10.808–14.473)	16.170 (13.850–18.794)	
≥1, <3	32.952 (30.924–35.046)	32.798 (29.740–36.010)	31.574 (28.611–34.694)	34.525 (31.743–37.417)	
≥3	52.684 (50.127–55.227)	52.744 (48.969–56.487)	55.900 (52.351–59.390)	49.305 (45.510–53.108)	
BMI categorical					<0.0001
≤18.5	1.598 (1.256–2.031)	2.111 (1.467–3.028)	1.492 (0.987–2.249)	1.213 (0.798–1.841)	
>18.5, ≤25	30.903 (29.000–32.873)	34.672 (31.823–37.636)	32.116 (29.449–34.906)	26.009 (23.251–28.971)	
>25, ≤30	30.524 (28.711–32.400)	33.150 (30.458–35.957)	29.931 (27.032–33.001)	28.601 (25.935–31.424)	
>30	36.975 (34.988–39.007)	30.067 (27.083–33.231)	36.460 (33.375–39.661)	44.177 (41.012–47.390)	
Anti-HBS					0.0091
(−)	51.172 (49.509–52.833)	50.148 (46.934–53.361)	48.721 (46.183–51.265)	54.692 (51.863–57.491)	
(+)	48.828 (47.167–50.491)	49.852 (46.639–53.066)	51.279 (48.735–53.817)	45.308 (42.509–48.137)	

For continuous variables, the survey-weighted mean (95% CI) and the *p*-value were measured using the survey-weighted t-test.

For categorical variables, such as survey-weighted percentage (95% CI), the *p*-value was determined using the survey-weighted Chi-square test.

PIR, poverty-to-income ratio; BMI, body mass index; SII, systemic immune-inflammation index; Q, tertiles.

### 3.2 Effects of the SII on seropositivity after hepatitis B vaccination

As shown in Table 3, increases in the SII and the Ln-transformed SII (LnSII) were negatively correlated with anti-HBS positivity after hepatitis B vaccination in both the crude model and Model 1 (OR = 0.9997, 95% CI = 0.9995–0.9999; OR = 0.868, 95% CI = 0.780–0.966). In Model 2, the negative correlation between LnSII and anti-HBS remains −0.983. However, in Model 2, the negative correlation between the SII and anti-HBS became insignificant (OR = 0.9998, 95% CI = 0.9996–1.0000). To assess the independent impact of the SII on antibody expression following hepatitis B vaccination, we converted the SII from a continuous variable to a categorical variable and constructed a correlation model for sensitivity analysis. Compared with a low-level SII, a high-level SII significantly reduced the incidence of serum HBsAb positivity in the crude model (OR = 0.8299, 95% CI = 0.7335–0.9391). After multivariable adjustment, the results remained robust and statistically significant (Model 1: OR = 0.7860, 95% CI = 0.6906–0.8947; Model 2: OR = 0.8661, 95% CI = 0.7577–0.9899). All of the above trends were statistically significant (*P* for trend <0.05).

**Table 3:** The association between SII and anti-HBS [Weighted ORs (95%CIs)].

	Model 0	Model 1	Model 2
SII	0.9997 (0.9995–0.9999)^**^	0.9996 (0.9994–0.9998)^***^	0.9998 (0.9996–1.0000)
Ln (SII)	0.868 (0.780–0.966)^**^	0.827 (0.739–0.925)^***^	0.876 (0.781–0.983)^*^
SII Tertile			
Low	1.0	1.0	1.0
Middle	0.9633 (0.8518–1.0894)	0.9241 (0.8130–1.0503)	0.9470 (0.8305–1.0798)
High	0.8299 (0.7335–0.9391)^**^	0.7860 (0.6906–0.8947)^***^	0.8661 (0.7577–0.9899)^*^
P for trend	0.0031	0.0003	0.0349

*p*-value was by survey-weighted regression. *p < 0.05; **p < 0.01; ***p < 0.001.

Model 1 was adjusted for age, sex, and race. Model 2 was adjusted for age, sex, race, PIR, BMI, education, alcohol status, smoking, and diabetes.

PIR, poverty-to-income ratio; BMI, body mass index; SII, systemic immune-inflammation index; LnSII, Ln-transformed SII.

### 3.3 Non-linear association

**Figure 2:**
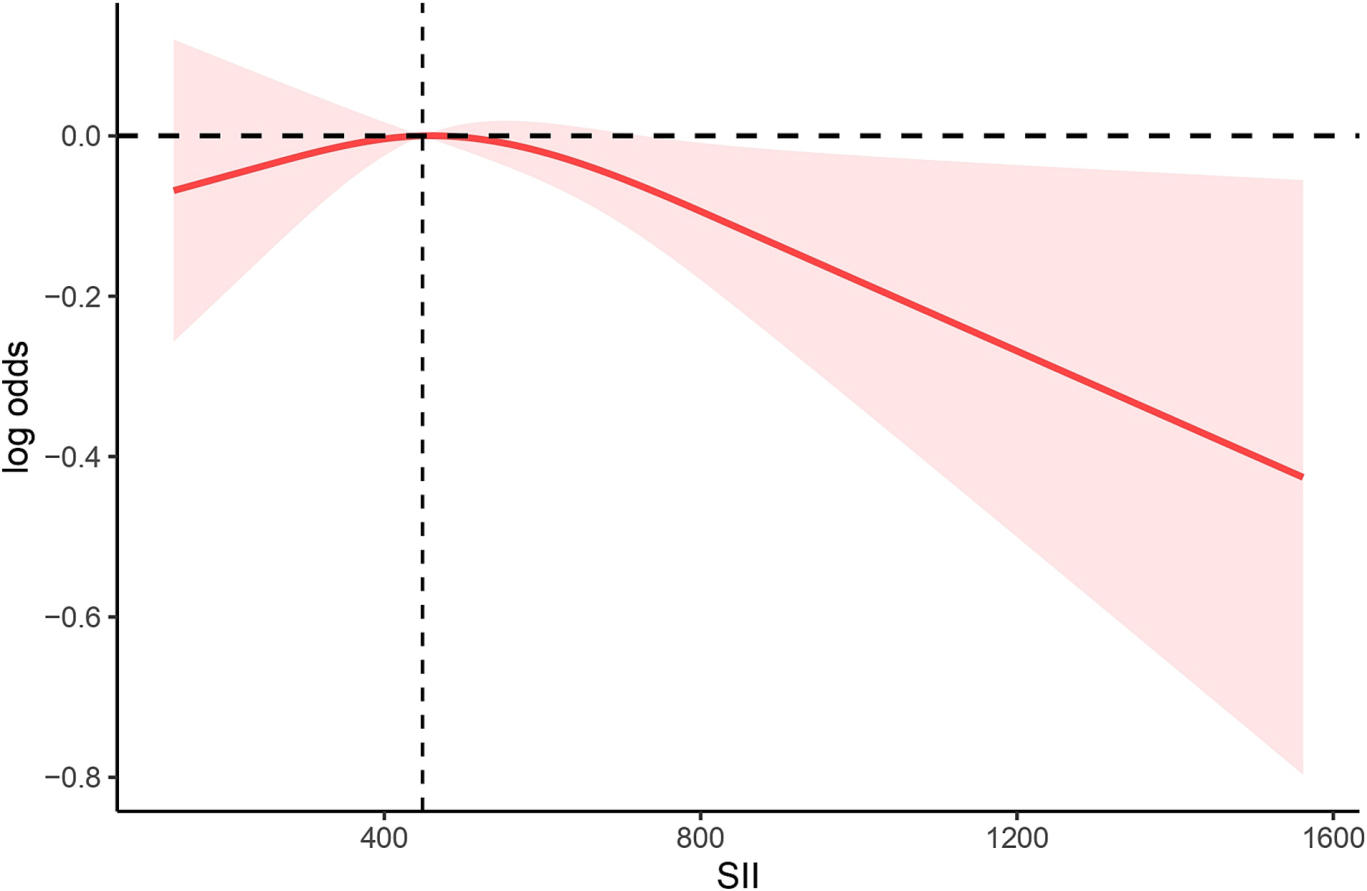
Spline plot of SII level and anti-HBS. The adjusted odd ratios and 95% confidence intervals (CIs) were calculated with logistic regression models after adjusting for age, sex, race, PIR, BMI, education, alcohol status, smoking, and diabetes.

We conducted RCS analysis in the amended model (Figure 2) to more precisely define the link between the SII and anti-HBS expression following hepatitis B immunization. Our RCS was set to 3, which is typically a good starting point for revealing complex non-linear relationships between variables without overfitting the data. Our findings revealed that there was no non-linear association between the SII and the hepatitis B vaccine’s antibody response (*p* = 0.1424). In regions where the SII was less than 448.3, the rate of negative correlation was relatively gentle, indicating that below this threshold, the antibody response is not particularly sensitive to changes in the SII. However, when the SII exceeded 448.3, the rate of negative correlation significantly increased, suggesting that high SII values may have a stronger negative impact on the antibody response. The threshold of approximately 448.3 indicates that, within this range of SII values, there is a stable relationship between the antibody response and the SII. Overall, the analysis reveals a predominantly linear negative correlation between the SII and anti-HBs antibody levels, particularly in the region where the SII values are above the identified threshold of 448.3.

### 3.4 Subgroup analyses and sensitivity analyses

**Figure 3:**
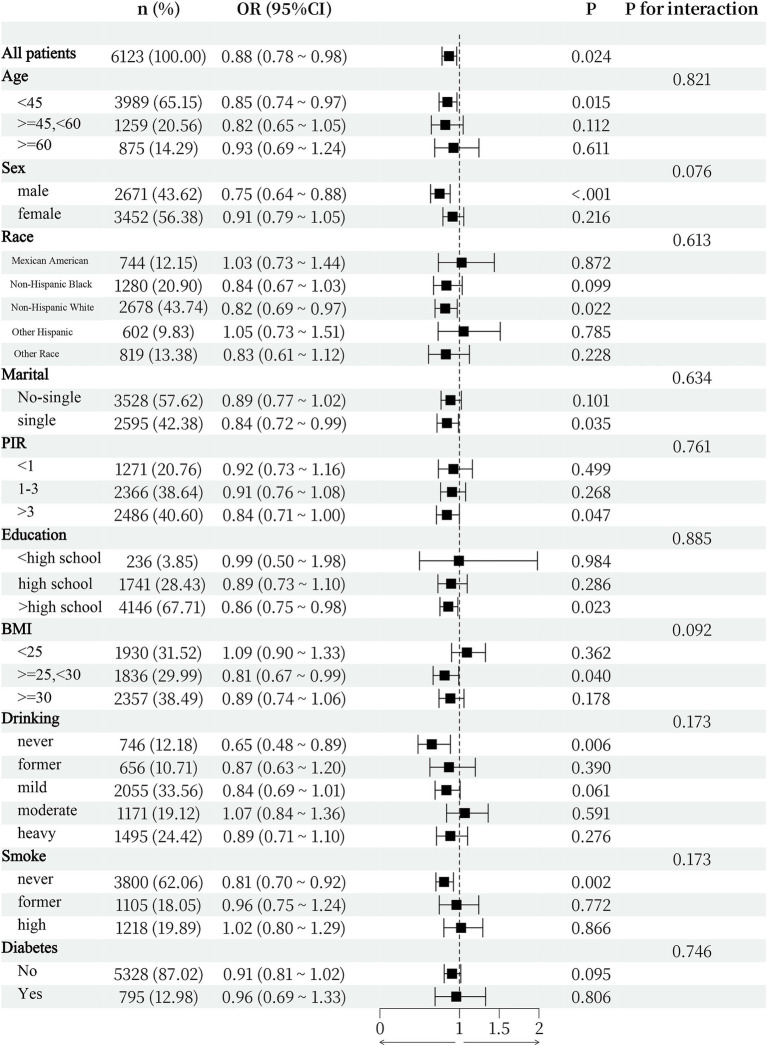
Subgroup analysis for the association between SII and anti-HBS.

As shown in Figure 3, subgroup analyses were conducted to determine whether demographic characteristics and comorbidities could account for the association between the two variables. The interaction test revealed that there was no statistically significant difference in the connection between the SII and the expression of anti-HBS across strata. This study demonstrated that the negative connection was not significantly influenced by age, sex, race, BMI, smoking status, drinking status, diabetes status, or other characteristics (interaction *p* > 0.05).

In our sensitivity analysis, we first categorized the SII into quartiles and quintiles to further assess its impact on anti-HBs (Supplementary Tables 1, 2). We observed that, in Model 1, regardless of whether the SII was classified into quartiles or quintiles, the OR for the highest level was frequently lower than that for the lowest level of the SII (OR = 0.813, 95% CI = 0.700–0.944; OR = 0.806, 95% CI = 0.682–0.952). In Model 2, this trend persisted; however, it did not reach statistical significance. Additionally, because the SII is calculated from lymphocytes, neutrophils, and platelets, we conducted a subgroup analysis. As shown in Table 4, after multivariable adjustment, only high-level neutrophils were negatively correlated with anti-HBS compared with low-level neutrophils (OR = 0.839, 95% CI = 0.731–0.964). Finally, owing to substantial disparities in variables and subject numbers between positive and negative populations for anti-HBS, we performed a 1:1 propensity score matching (PSM) analysis on the basis of factors that may influence immunity, such as sex and age. We then performed a correlation analysis between the SII and anti-HBs (Supplementary Table 3). We found that LnSII and high SII values were negatively correlated with anti-HBS. All of the above trends were statistically significant (*P* for trend <0.05).

**Table 4:** The association between SII and lymphocyte, neutrophil, and platelet.

	Non-adjusted	Model 1	Model 2
L	0.920 (0.856–0.989)*	0.846 (0.784–0.913)***	0.950 (0.877–1.028)
L tertile			
Low	1.0	1.0	1.0
Middle	1.013 (0.893–1.150)	0.941 (0.825–1.073)	1.025 (0.895–1.174)
High	0.912 (0.808–1.029)	0.796 (0.701–0.904)***	0.961 (0.841–1.098)
P for trend	0.12669	0.00037	0.54194
N	0.931 (0.903–0.961)***	0.911 (0.881–0.941)***	0.970 (0.936–1.005)
N tertile			
Low	1.0	1.0	1.0
Middle	0.883 (0.781–0.999)*	0.833 (0.732–0.948)**	0.900 (0.789–1.028)
High	0.739 (0.653–0.836)***	0.676 (0.593–0.770)***	0.839 (0.731–0.964)*
P for trend	<0.00001	<0.00001	0.01343
PLT	1.000 (0.999–1.000)	0.998 (0.998–0.999)***	0.999 (0.998–1.000)
PLT tertile			
Low	1.0	1.0	1.0
Middle	1.037 (0.917–1.174)	0.930 (0.818–1.057)	0.940 (0.823–1.072)
High	0.976 (0.862–1.105)	0.834 (0.730–0.952)**	0.915 (0.797–1.050)
P for trend	0.69432	0.00702	0.20526

*p*-value was by survey-weighted regression. *p < 0.05; **p < 0.01; ***p < 0.001.

Model 1 was adjusted for age, sex, and race. Model 2 was adjusted for age, sex, race, PIR, BMI, education, alcohol status, smoking, and diabetes.

PIR, poverty-to-income ratio; BMI, body mass index; L, Lymphocyte; N, Neutrophils; PLT, Platelet.

## 4 Discussion

Hepatitis B virus infection is a global public health problem with high morbidity and mortality. The greatest method for preventing and limiting the spread of HBV is the safe and effective hepatitis B vaccine. Nevertheless, some people who receive the hepatitis B vaccine are unable to respond appropriately. We investigated the relationships between the SII, a marker of the inflammatory state and immune response, and the hepatitis B vaccine antibody response, given that sex and age affect the antibody response. This cross-sectional investigation verified that the SII is associated with a weakened immune response after hepatitis B vaccination. The findings from the subgroup analysis, sensitivity analysis, and interaction tests collectively suggest that the observed correlation is pervasive across the studied population.

Abnormal expression of systemic inflammatory markers may reflect immune dysfunction in the body. The levels of plasma IP10, IL-6, sCD14, and sCD163 before vaccination were found to be negatively correlated with the antibody responses to HAV, HBV, and tetanus after vaccination in a case–control study where the levels of the plasma soluble inflammatory index were measured in subjects before and after vaccination (26). Weinberger investigated the impact of age on primary and recall antibody responses following hepatitis B vaccination in young and older adults. They reported that, after primary vaccination, antibody responses were lower and delayed in older adults than in young adults. Transcriptome analysis revealed that proinflammatory pathways are predominant in older adults, which contributes to their reduced responsiveness to vaccines (27). This discovery is in agreement with the findings of the study by Fourati et al., who used transcriptional and cytometric profiling of whole blood collected before vaccination and reported that higher levels of inflammatory response transcripts and increased frequencies of proinflammatory innate cells correlate with weaker responses to hepatitis B vaccination (28).

Furthermore, the induction effect of the proinflammatory cytokine IL-15 has been validated at both the gene and protein levels in individuals who are low responders to the HBV vaccine. Proinflammatory complement complexes such as C3 and VEGF (a vascular endothelial growth factor that promotes chronic inflammation) are also negatively correlated with antibody production levels. In contrast, low responders to the hepatitis B vaccine had anti-inflammatory protein-coding genes such as CD200 and BATF3 silenced (28). In contrast, anti-inflammatory protein-coding genes such as CD200 were silenced in low responders to the hepatitis B vaccine.

SII, a marker of immunological and inflammatory activity, has been confirmed to be associated with the onset of several autoimmune diseases, such as rheumatoid arthritis (29) and psoriasis (30), indicating that the SII may affect the body’s immune function. The SII is derived from neutrophils, lymphocytes, and platelets. Therefore, changes in the quantity or function of lymphocytes, neutrophils, or platelets may act as a mediator between the SII and the poor production of antibodies following hepatitis B vaccination. An increase in the SII is associated with either increased neutrophil and platelet counts or decreased lymphocyte counts. The immune response is regulated by lymphocyte subsets, which can induce or inhibit humoral and cellular immunity; thus, the non-response to the hepatitis B vaccine is considered to be related to impaired lymphocyte activation (31). An earlier study revealed that the proportion of helper T cells in people who did not respond to the hepatitis B vaccine significantly decreased before vaccination, and it was hypothesized that determining the peripheral blood lymphocyte subset count prior to vaccination might help determine how well the vaccine would respond to the hepatitis B vaccine (32). Neutrophils have also been found to act as immunosuppressants by inhibiting the proliferation and activation of T cells (33). Platelets, on the other hand, regulate innate and adaptive immunity and accelerate the inflammatory state by interacting with monocytes, neutrophils, and lymphocytes (34).

The production of several inflammatory mediators has also been linked to immune-mediated tissue damage, which can accelerate the progression of cirrhosis and liver cancer in hepatitis B patients (35, 36). Child–Pugh, ALB, total bilirubin, ALP, and prothrombin time are traditional markers of liver injury that are strongly connected with the systemic inflammatory response index (SIRI), with a higher SIRI indicating worse liver function. Neutrophil infiltration is associated with liver inflammation and subsequent inflammation-induced pathological damage (37). Neutrophils are thought to play a significant role in liver damage and healing during the advanced stages of cirrhosis, increasing the risk of infection and organ failure. Platelet activation is related to viral and bacterial hepatitis (38). Serotonin produced by platelets can worsen viral hepatitis. On the other hand, the absence of platelet-derived serotonin can ameliorate hepatic microcirculation dysfunction, accelerate the clearance of liver viruses, and reduce CD8^+^ T-cell-dependent liver damage (39). Owing to the body’s inadequate immune function and increased susceptibility to secondary infections caused by this high inflammatory state, increased organ malfunction and mortality will eventually result (40). Therefore, the SII may reduce the immune response of the body to the hepatitis B vaccine by reducing immune cell function through its high inflammatory state. The PLR and NLR are strongly related to the severity of HBV infection and partially represent the amount of blood HBV DNA and serum HBeAg in individuals with CHB (41). An independent predictor of mortality in patients with HBV infection, the NLR value was reported to be substantially greater in patients with hepatitis B than in healthy individuals in a case–control investigation (42). The SII can provide more clinical information than just one or two types of peripheral blood because of its unique calculation process. In a population-based cohort analysis of 196 Indonesian patients with advanced HCC, the SII outperformed the NLR in predicting the 1-year survival rate of untreated patients (43).

Individual immunological health has a significant effect on how much antibody is produced. For debate, we grouped congenital individual variations, such as sex, age, and race, together. In terms of sex, the basic level of immunoglobulin (44) and the antibody response to viruses and vaccines (45) in women are always greater than those in men. After hepatitis B vaccination, the rate of serum antibody positivity in adult women was significantly greater than that in men (46), which is consistent with the findings of this study. This result may be because there are related immune genes on the X chromosome, while there are few related immune genes on the Y chromosome (47). In addition, oestrogen can activate monocytes to secrete IL-10, which in turn induces B cells to secrete IgG and IgM (48), whereas testosterone significantly reduces the levels of IgG and IgM produced by B lymphocytes (49). In terms of age, we discovered that people who responded to vaccination tended to be younger, which is possibly related to the body’s immune system deteriorating with age. The immune response may be affected by thymus degradation, variations in cytokine production or distribution, and variations in the quantity or quality of lymphocyte populations (50). Previous studies have revealed significant racial differences in the percentage of people who do not respond to the hepatitis B vaccine, which may be connected to differences in the environment, mutation rate, and genetic variability (21, 51).

High BMI, diabetes, and smoking were all features of those with poor anti-HBS expression in our study. The vaccine is mostly distributed in fat rather than muscle, which may inhibit the absorption of the vaccine and denature it by enzymatic action, which may account for the low response of overweight people after vaccination (52). Numerous studies have also shown that obesity can lead to inflammation, which is distinct from other types of inflammation in that it includes the activation of the innate immune system. Obesity-induced inflammation can lead to an increase in the number and activation of immune cells and the production of proinflammatory cytokines while inhibiting the production of anti-inflammatory cells, increasing the susceptibility of the body to various cellular stresses, such as endoplasmic reticulum stress and mitochondrial dysfunction (53), which can affect metabolic homeostasis (54). The aetiology of obesity-related insulin resistance and type 2 diabetes is linked to persistent low-grade inflammation and immune system activation (55). Currently, the positive rate of hepatitis B antibodies in diabetic patients is lower than that in healthy individuals. The percentage of positive serum samples gradually decreased from the normal glucose tolerance group (53.64%), to the abnormal glucose tolerance group (45.52%), and then to the diabetic group (28.84%, *p* < 0.0001). The relatively low levels of antibody production in smokers may be caused by nicotine-mediated inhibition of the B cell response through the destruction of the antigen-mediated pathway and the intracellular calcium response in T cells. Smoking status and BMI are also positively related to the NLR, PLR, and SII (56, 57).

The changes in antibody levels are not only related to individual immune status and the regulation of systemic inflammatory responses but are also closely associated with the time interval after vaccination. Therefore, when conducting vaccine-related research, it is essential to pay attention to the impact of time on antibody titres. A single-center study on the changes in HBV antibody titres over time in children revealed that HBsAb titres in children decrease over time, with 50% of children over 7 years of age being seronegative and HBsAb titres reaching zero by 13 years of age. This finding indicates that HBsAb titres continuously decline over time (58). A cross-sectional survey from rural China revealed that, after 20–31 years of primary vaccination with a plasma-derived hepatitis B vaccine, the average positive rate of anti-HBs was 53.21%, and a decreasing trend was observed (59). However, a retrospective study by Bruce et al. revealed that among the population who experienced an antibody response after initial hepatitis B vaccination, 51% maintained high antibody levels 30 years later. In contrast, among those who did not respond to the initial vaccination, 88% were able to produce sufficient antibody levels after receiving one booster dose of recombinant hepatitis B vaccine. Therefore, they estimated that ≥90% of participants had evidence of protection 30 years later and that booster doses are not needed (60). In summary, time is crucial for changes in antibody titres following vaccination.

Research on the relationship between the SII and the antibody response to the hepatitis B vaccine is undoubtedly in the pioneering stage of exploration. However, we must recognize that there are several limitations in the current research, which necessitate further in-depth studies in the future. First, this study employed a cross-sectional design, which cannot reveal the causal relationship between the SII and the antibody response to the hepatitis B vaccine. Although our findings indicate that individuals with high SII values are less likely to produce effective antibodies after vaccination, these observations are merely descriptive of an association and do not establish causality. Second, owing to the inability to accurately record the specific time from vaccination to the measurement of serological markers in the study, we may not have been able to accurately assess the seropositivity rate. This temporal ambiguity could have led to the loss of some key information, thereby affecting our evaluation of the vaccine’s effectiveness. We also cannot determine whether the impact of the SII on hepatitis B antibodies is transient or has a lasting effect over time. Third, this study is limited by the constraints of data collection and does not cover the patients’ medication status. Medications may influence the production of vaccine antibodies and the immune response process, making this an important factor that we cannot overlook. Therefore, future prospective cohort studies should be conducted to better understand the causal relationship between the SII and immune responses to the hepatitis B vaccine. Additionally, increasing the sample size and population diversity, along with employing longitudinal designs to regularly monitor changes in the SII, will help reveal the potential mechanisms by which the SII influences immune responses following vaccination, providing important evidence for personalized vaccination strategies.

## 5 Conclusion

This study is the first to explore the relationship between the SII and the antibody response to the hepatitis B vaccine. The SII value in the group that did not produce sufficient antibodies was significantly greater than that in the group that did produce sufficient antibodies, and there was a linear negative correlation between the two variables. This result suggests that high SII values may be associated with a poor antibody response to the hepatitis B vaccine. Future rigorous experiments are needed to further confirm this association and to determine its potential mechanisms.

## Data Availability

Publicly available datasets were analyzed in this study. This data can be found here: https://wwwn.cdc.gov/nchs/nhanes/Default.aspx.

## Footnotes

^1^ https://wwwn.cdc.gov/nchs/nhanes/Default.aspx
