# Supplementary material for: Association between the systemic immune-inflammatory index and the immune response after hepatitis B vaccination: a cross-sectional analysis of NHANES data
Source: Front Public Health. 2025 May 14;13:1480766. doi: 10.3389/fpubh.2025.1480766 (PMC12116451; doi:10.3389/fpubh.2025.1480766)
Supplement: Supplementary file 1 [file Data_Sheet_1.docx]

Supplementary Table 1. The association between SII and anti-HBS [ Weighted ORs (95%CIs)] (Classified by SII quartiles).

|  | Model 0 | Model 1 | Model 2 |
| --- | --- | --- | --- |
| SII quartile |  |  |  |
| Q1 | 1.0 | 1.0 | 1.0 |
| Q2 | 1.039 (0.901, 1.197) | 0.994 (0.858, 1.152) | 0.986 (0.848, 1.146) |
| Q3 | 0.907 (0.787, 1.046) | 0.843 (0.726, 0.978) ^*^ | 0.855 (0.734, 0.995) ^*^ |
| Q4 | 0.865 (0.750, 0.997) ^*^ | 0.813 (0.700, 0.944) ^***^ | 0.873 (0.749, 1.017) |
| P for trend | 0.0130 | 0.0011 | 0.0257 |

*P*-value was by survey-weighted regression. ^*^*P*<0.05; ^**^*P* <0.01; ^***^*P* <0.001

Model 1 was adjusted for age, sex, race. Model 2 was adjusted for age, sex, race, PIR, BMI, education, alcohol status; smoke, diabetes.

Supplementary Table 2. The association between SII and anti-HBS [ Weighted ORs (95%CIs)] .( Classified by SII quintiles)

|  | Model 0 | Model 1 | Model 2 |
| --- | --- | --- | --- |
| SII 5 quantiles |  |  |  |
| Q1 | 1.0 | 1.0 | 1.0 |
| Q2 | 1.042 (0.889, 1.221) | 1.011 (0.857, 1.192) | 1.020 (0.862, 1.206) |
| Q3 | 0.931 (0.794, 1.092) | 0.870 (0.737, 1.027) | 0.878 (0.742, 1.040) |
| Q4 | 0.869 (0.741, 1.018) | 0.804 (0.681, 0.950) ^*^ | 0.829 (0.700, 0.983) ^*^ |
| Q5 | 0.856 (0.730, 1.004) | 0.806 (0.682, 0.952) ^*^ | 0.874 (0.737, 1.037) |
| P for trend | 0.0067 | 0.0005 | 0.0148 |

*P*-value was by survey-weighted regression. ^*^*P*<0.05; ^**^*P* <0.01; ^***^*P* <0.001

Model 1 was adjusted for age, sex, race. Model 2 was adjusted for age, sex, race, PIR, BMI, education, alcohol status; smoke, diabetes.

Supplementary Table 3. The association between SII and anti-HBS after PSM [ Weighted ORs (95%CIs)] .

|  | Statistics | Model 0 |
| --- | --- | --- |
| SII | 498.626 ± 243.322 | 1.000 (1.000, 1.000) |
| Ln (SII) | 6.102 ± 0.470 | 0.886 (0.828, 0.936) ^***^ |
| SII tertile |  |  |
| Low | 1847 (33.339%) | 1.0 |
| Middle | 1846 (33.321%) | 1.005 (0.884, 1.144) |
| High | 1847 (33.339%) | 0.876 (0.770, 0.997) ^*^ |
| P for trend |  | 0.0447 |

*P*-value was by survey-weighted regression. ^*^*P*<0.05; ^**^*P* <0.01; ^***^*P* <0.001
